# First-principles Investigations of Magnetic Semiconductors: An example of Transition Metal Decorated Two-dimensional SnS Monolayer

**DOI:** 10.3390/nano8100789

**Published:** 2018-10-04

**Authors:** Fangfang Wang, Liyu Zhou, Zhen Ma, Mingxue He, Fang Wu, Yunfei Liu

**Affiliations:** 1 College of Information Science and Technology, Nanjing Forestry University, Nanjing 210037, China; wangff100@gmail.com (F.W.); maxwell070311@gmail.com (L.Z.); mazhen011@gmail.com (Z.M.); hemingxue370@gmail.com (M.H.); lyf@njfu.com.cn (Y.L.); 2 Institution of Energy and Microstructure, Nanjing University of Science and Technology, Nanjing 210094, China

**Keywords:** density functional theory, magnetic semiconductors, doping

## Abstract

The absence of magnetic moments in pristine two-dimensional (2D) semiconducting materials has attracted many research interests. Transition-metal (TM) decoration has been found to be an effective strategy to introduce magnetic moments in non-magnetic 2D semiconductors. However, the stability of TM atoms modified 2D semiconductors has not been well explored. Here, taking 2D Tin (II) sulfide (SnS) monolayer as a prototype, we explored the stability of magnetic semiconductors through this method. In our studies, all possible configurations of TM decoration have been considered, namely, adsorption on the intact surface, S vacancy, and Sn vacancy. Based on the energy gain and electronic analysis, our results revealed that most of the TM atoms will form a cluster, and only several TM atoms can be effectively doped into the SnS monolayer. Furthermore, the band calculations showed that only Mn substitution will give rise to a magnetic semiconductor. Thus, the reported results here provide some hidden information for further realization of the magnetic semiconductors and serve as a paradigm to prepare 2D magnetic semiconductors.

## 1. Introduction

Two-dimensional (2D) layer structured materials have received great research interests, particularly its peculiar physical properties due to their low dimensionality and electron confinement [1,2,3,4,5]. In the past few years, extensive investigations have been focused on graphene and similar 2D nanomaterials, such as hexagonal boron nitride (h-BN) sheet, transition metal dichalcogenide and MXenes, as they have potential applications in the next-generation electric or photonic devices [6,7,8,9,10]. However, such pristine 2D materials cannot satisfy the requirements of real applications with their intrinsic properties. This has led to the electronic, magnetic and mechanic properties with feasible solutions to be one of the most commonly discussed topics in this field.

On the other hand, magnetic semiconductors are one of the functional materials exhibiting magnetism and semiconductor properties, which can significantly improve the performance of electric devices. Developing practical magnetic semiconductors have been listed as one of 125 scientific frontier issues, announced by the Science magazine [11]. Since 2D materials have been taken as the candidates in the next-generational electric devices, developing 2D magnetic semiconductors become more and more important. However, the most widely studied 2D semiconductors are nonmagnetic. Previously, numerous studies have demonstrated that the magnetic moments can be induced by various factors such as edge states, vacancy defect, adatom defect or substitutional doping [12,13,14]. For example, Krasheninnikov et al. and Wei et al. suggested that by doping transition metal (TM) into the lattice of boron nitride monolayer, such materials can get to the spin-polarized state [7,15]. Thus, such a method may provide a solution to introduce magnetic moments in 2D semiconductors.

However, most of the 2D semiconductors are quite stable because of their strong bonding within the layer. Therefore, it is necessary to answer whether TM atoms can be effectively doped into 2D precursors. Furthermore, although most of TMs are magnetic elements, their participation in the 2D semiconductors may create new electronic states and annihilate the spin moments. Thus, theoretical understanding at the atomic scale is necessary to provide enough physical pictures for developing 2D magnetic semiconductors.

Recently, a new semiconductor material named herzenbergite SnS has attracted great research interests. Herzenbergite SnS is comprised of earth abundant elements and is relatively nontoxic [16]. The crystal structure of herzenbergite SnS is a layered structure of strong Sn-S bonds within a puckered sheet with weak intermolecular interactions between the layers, which is similar to phosphene [17,18,19]. The bulk of SnS has an indirect band gap with a large absorbance coefficient (α > 10^4^) across the ultraviolet visible and near-infrared regions of the electromagnetic spectrum, making it potentially applicable in photovoltaic devices [20,21]. To get the 2D SnS monolayer, many previous works have been reported in experiments [16,22,23,24]. Based on such experimental results, theoretical works have been performed to study the electronic, optical and other properties [25,26,27,28,29,30,31,32,33,34].

Here, taking 2D SnS as an example of low-dimensional semiconductors, we explored the intrinsic mechanism of TM atoms interacting with 2D semiconductors. Through the first-principles calculations and total energy analysis, we showed that only Sc, Ti, Mn and Zn can be effectively doped into SnS matrix, which is quite different with the general understanding. Also, the electronic calculations showed that only Mn-doped SnS is a magnetic semiconductor with a large net spin moment. Thus, our results indicate that not all the TM atoms can be doped into semiconductors, and choosing suitable elements is the key to developing 2D magnetic semiconductors.

## 2. Methods

Our first-principles calculations were based on density functional theory (DFT) implemented in the Vienna ab initio simulation package (VASP) code [35]. For the exchange-correlation energy, the Perdew, Burke, and Ernzerhof (PBE) functional [36] and the spin-polarized hybrid Heyd-Scuseria-Ernzerhof (HSE) functional [37,38] were used for our research. The projected augmented wave (PAW) method with a plane-wave basis set was used for the ion-electron interaction [39,40]. For the spin-polarized calculations, the Vosko-Wilk-Nusair modification scheme was applied to interpolate the correlation energy [41].

We chose a 5 × 5 supercell along the *y* and *z* directions of the 2D SnS monolayer. Thus, with this structure, we adsorbed single TM atoms on the pristine 2D SnS monolayer, and TM atoms were embedded in a single S atom vacancy or substituted a single Sn atom of 2D SnS monolayer, which were denoted as TM@P-SnS, TM@V_S_-SnS and TM@S_sn_SnS, respectively (TM = Co, Cr, Cu, Fe, Mn, Ni, Sc, Ti, V and Zn). The electron correlation effect may play a role in magnetic properties of TM elements due to the localized d-orbital. Therefore, we also carried out the GGA+U calculations with U = 4 eV to check the magnetic state of TM-doped systems [42]. In the direction perpendicular to the 2D SnS monolayer, a vacuum layer of 30 Å was used to keep the spurious interaction between neighboring slabs negligible. A 450 eV cutoff was used for our research. All of the structures were relaxed using the conjugate gradient method, and the convergence criterions were set to be 10^−4^ eV in energy and 10^-3^ eV/Å in force. The Brillouin zone was represented by Monkhorst-Pack special *K*-point mesh [43,44] of 1 × 3 × 3 during the geometry optimization, while a 1 × 5 × 5 *K*-point was used for electronic structure calculations.

## 3. Results and Discussion

**Figure 1:**
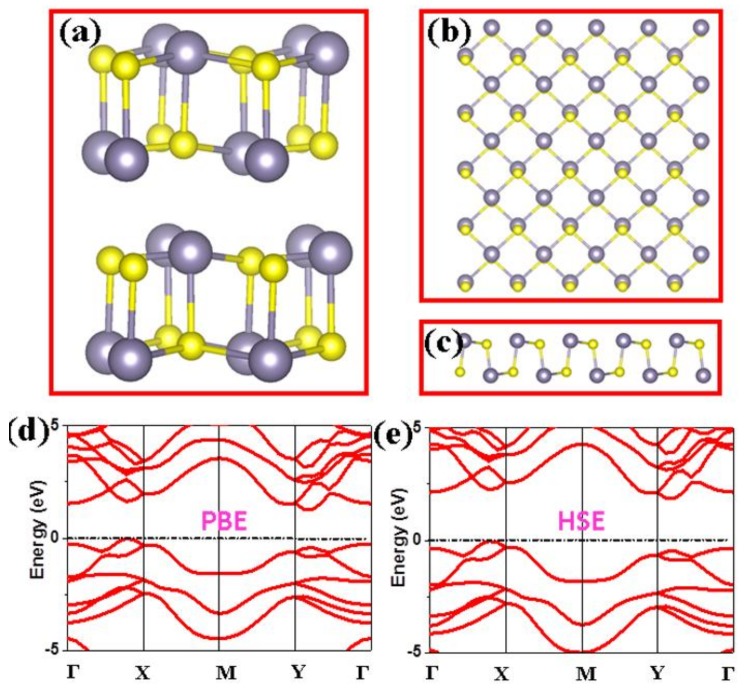
The crystal structures for (**a**) 3D bulk SnS; (**b**) top view of 2D SnS monolayer; (**c**) side view of 2D SnS monolayer; The calculated band structure of 2D monolayer with PBE (**d**) and HSE (**e**). The Γ-Χ-Μ-Υ-Γ means the high symmetric points in Brillouin zone.

Similar to the black phosphorus, the crystal structure of 3D bulk SnS had strong Sn-S bonds within a puckered sheet and weak intermolecular interactions between the layers, as shown in Figure 1a. When the 2D honeycomb SnS monolayer mechanically exfoliated from the layered 3D bulk, the puckered structure within the sheet was well kept, as shown in Figure 1b,c. Moreover, we calculated the band structures of the 2D SnS monolayer by using the PBE and HSE functional, and found that all atoms of 2D SnS monolayer had no magnetic moments. As depicted in Figure 1d, the calculated band structures show that the bands of the spin-up channel are exactly the same with that of the spin-down channel, which agrees with the others’ results [25,26,27,28,29,30,31,32,33,34]. Thus, we can conclude that 2D SnS monolayer is a nonmagnetic semiconductor with an indirect band gap (as shown in Figure S1). According to the band structures based on DFT calculations, the values of the band gap obtained from the PBE and HSE are about 1.4 eV and 2.0 eV, respectively. It is well known that the PBE usually underestimates the band gap while the HSE can show a better agreement with the previous theoretical and experimental results [16,45,46,47].

**Figure 2:**
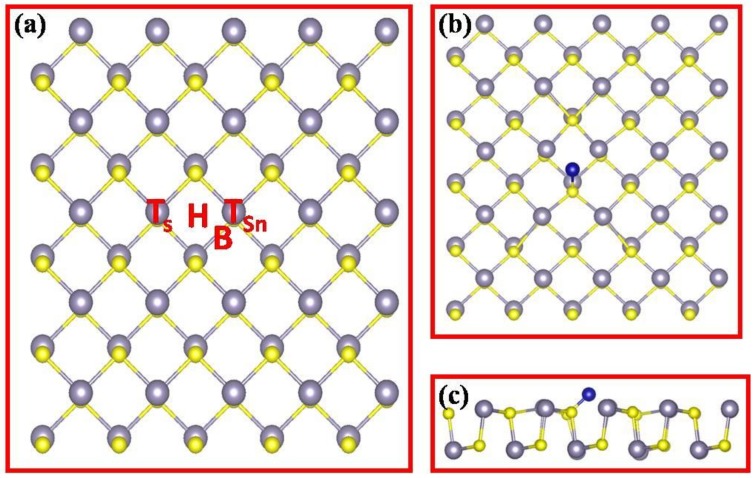
(**a**) Four possible sites of transitional metal atoms doping on 2D SnS pristine monolayer: the top site of a S atom (T_S_) or on a Sn atom (T_Sn_), the bridge site over a S-Sn bond (**b**), and the hollow site of a hexagon ring (H); (**b**) The top view of the most stable state; (**c**) The side view of the most stable state.

Previous research of diluted magnetic semiconductors found that doped TM atoms can easily form other impurity phases, which could lead to the inconsistent results [48]. Thus, it is natural to question whether TM atoms can be effectively doped into the SnS monolayer. To do so, all the possible configurations of TM decorated SnS were considered in our research. First of all, we considered the possibility of direct adsorption of TM atoms on the pristine SnS monolayer (TM@P-SnS). As shown in Figure 2a, four possible adsorption sites of TM atoms were listed: (1) the top site of a S atom (TS) or (2) on a Sn atom (TSn), (3) the bridge site over a S-Sn bond (B), and (4) the hollow site of a hexagon ring (H). Interestingly, we found that even if the TM atoms were initially put on the T or B sites, after structural relaxation, TM atoms spontaneously moved to the H site. Namely, only H site could be used to adsorb TM atoms when such TM atoms were directly deposited on 2D SnS monolayer. Figure 2b shows the final structure after geometric relaxations when TM atoms stay at the H site, which is consistent with the situation of phosphorene [49,50].

**Figure 3:**
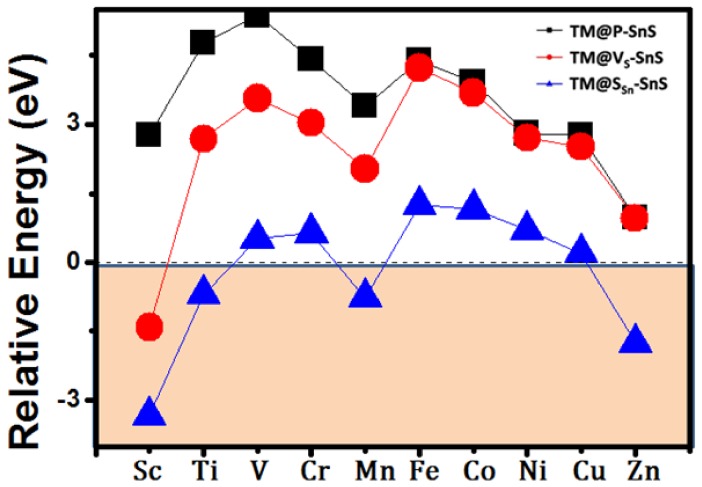
Relative energies of the TM decorated 2D SnS monolayer, which is defined as the energy difference of binding energy of TM on the SnS monolayer and the cohesive energies of TM atoms. A negative value of relative energy indicates that TM atoms will not form a cluster, and the systems are stable.

The binding energies (*E*_b_) for the TM atoms on the 2D SnS monolayer are defined as *E_b_* = *E*_TM@P-SnS_ − *E*_P-SnS_ − *E*_TM_, where *E*_P-SnS_, *E*_TM_, and *E*_TM@P-SnS_ are the total energies of the pristine 2D SnS monolayer, the isolated TM atom, and the TM@P-SnS system, respectively. Since H site is preferred by the TM atoms, we only focused on such situations in the following research. To judge whether the adsorptive TM atoms will form a metal cluster, it is reasonable to compare the *E*_b_ with the cohesive energies of TM atoms. If the binding energy is larger than the cohesive energies, it means that TM atoms will not form a TM cluster. Otherwise, TM atoms will spontaneously form a TM cluster. As presented in Figure 3, all the calculated binding energy of TM@P-SnS are much smaller than the cohesive energies of TM atoms. Thus, according to our calculations, direct deposition of TM atoms on the pristine SnS monolayer will lead to TM cluster, and cannot produce uniformed magnetic structures.

Since pristine SnS monolayer cannot strongly interact with TM atoms, we further consider TM atoms embedded in 2D SnS monolayer with a single S atom vacancy (TM@Vs-SnS). In this sense, the binding energies are defined as *E*_b_ = *E*_TM@Vs-SnS_ − *E*_Vs-SnS_ − *E*_TM_, where the *E*_TM@Vs-SnS_ is the total energies of TM atoms embedded 2D SnS monolayer with a single S atom vacancy, and *E*_Vs-SnS_ is the energy of 2D SnS monolayer with a single S atom vacancy. As an attracting site, S vacancy will capture the adsorptive TM atoms after geometric relaxations. Our total energy shows that the binding energy is slightly enhanced compared with the pristine SnS monolayer by introducing S vacancy (as shown in Figure S2). However, compared with the cohesive energies of TM atoms, such binding energy indicates that TM atoms will still form a cluster on the monolayer except for Sc atoms. Consequently, direct deposition of TM atoms on SnS monolayer cannot lead to controllable magnetic ordering.

Now, we consider the case that TM atoms substituting a single Sn atom in 2D SnS monolayer. Adopting the same method used in the TM@P-SnS and TM@Vs-SnS systems, we calculated the binding energy of different TM atoms. Our results showed that most of TM atoms are strongly bound in the framework of SnS monolayer with a large binding energy (ranging from −2.92 to −7.45 eV), suggesting that TM atoms strongly interact with the surrounding atoms. However, compared with the cohesive energies of TM atoms, only Sc, Ti, Mn and Zn can avoid the possibility of forming TM cluster, and be effectively doped into SnS monolayer, which can be experimentally realized through the alloy method.

**Figure 4:**
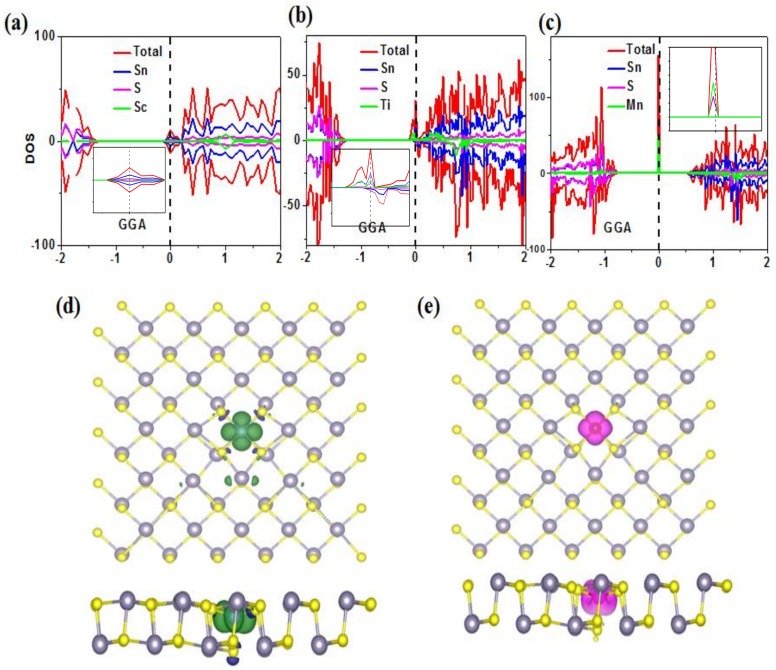
(**a**), (**b**) and (**c**) are the calculated partial density of states (PDOS) plot of the Sc-, Ti-, and Mn-substituting a Sn atom in SnS monolayer, respectively, the inset is the enlarged parts around Fermi level. The energy range (eV) of plotted PDOS is shown in X-axis (**d**) and (**e**) are the spin density plot for Ti- and Mn- substituting a Sn atom in SnS monolayer, respectively.

In the following research of the paper, we will focus on such TM atoms (Sc, Ti, Mn and Zn) substituting a Sn atom in SnS monolayer. Generally, the doped TM atoms will donate two electrons to the surrounding S ions which may lead to the local magnetic moments. The calculated spin moments show that Sc and Zn doped SnS prefer non-spin-polarized solutions, while Ti (Mn) doped SnS has a net magnetic moment of 1.9 (5) uB. It is not surprising that Zn-doped SnS has no net magnetic moment since the d orbitals are fully occupied. However, for Sc-doped SnS, Figure 4a shows that valence electrons strongly hybrid with the surrounding S atoms, which can lead to the non-spinpolarized electronic structures. For the Ti-doped SnS system, the calculated net spin moment was 1.9 uB, which indicates that two electrons in d orbitals are not fully localized. As shown in Figure 4b and 4d, both the partial density of state (PDOS) and the spin density plot show that valence electrons of Ti overlap with the surround S atoms. For the Mn-doped SnS system, the calculated net spin moment is 5 uB, which indicates that five electrons in d orbitals are fully localized.

**Figure 5:**
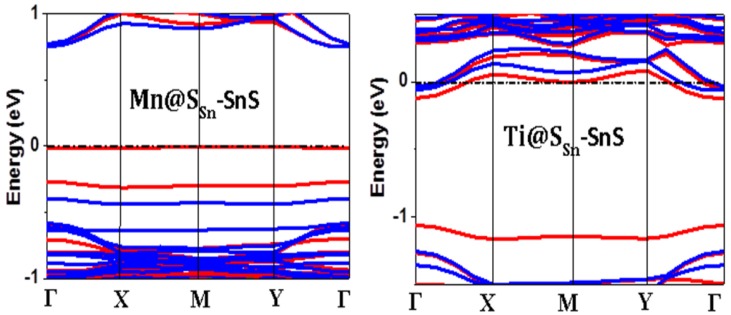
Band Structures of Mn-doped SnS and Ti-doped SnS with GGA + U calculations, where Γ-Χ-Μ-Υ-Γ means the high symmetric points in Brillouin zone.

Since only Ti and Mn-doped SnS are magnetic systems, we focused our following studies on such systems. In Figure 5, we present the calculated band structure by using the GGA + U method (Our GGA calculations give the same pictures, which are ignored here). As explored in PDOS and spin density plot, Ti-doped SnS monolayer shows metallic character, while Mn-doped SnS monolayer keeps the semiconducting character, which provides the possibility of preparing magnetic semiconductors.

On the other hand, the magnetic interaction between the doped Mn atoms is also quite important for practical applications. However, in such systems, there are many possible configurations of doped sites, making the calculation of magnetic interaction quite difficult. Since many groups have reported that generally adopting supercell models cannot correctly describe the real situations [51,52], new methods are necessary to discuss the magnetic interaction, which is beyond the scope of current manuscript.

## 4. Conclusions

In summary, a systematic study on the magnetic and electronic properties of the TM decorated 2D SnS monolayer has been carried out by using the first-principles calculations in the present work. Our results show that not all the TM atoms can be decorated on the SnS monolayer, only Sc, Ti, Mn and Zn atoms can be effectively doped into the framework of 2D SnS monolayer. Moreover, the calculated spin moments of such systems demonstrate that Ti and Mn atoms can induce local spin moments in the monolayer. The following band structure calculations determined that only Mn-doped SnS is a magnetic semiconductor, while Ti-doped SnS monolayer is spin-resolved metal.

## Supplementary Materials

The following are available online at http://www.mdpi.com/2079-4991/8/10/789/s1, Figure S1: The calculated spin-resolved density of states of 2D SnS monolayer, Figure S2: The calculated binding energy for each TM atom in 2D SnS monolayer.
