# Supplementary material for: First-principles Investigations of Magnetic Semiconductors: An example of Transition Metal Decorated Two-dimensional SnS Monolayer
Source: Nanomaterials (Basel). 2018 Oct 4;8(10):789. doi: 10.3390/nano8100789 (PMC6215100; doi:10.3390/nano8100789)
Supplement: Supplementary file 1 [file nanomaterials-08-00789-s001.pdf]

First-principles Investigations of Magnetic Semiconductors: An example of Transition Metal Decorated Two-dimensional SnS Monolayer

Fangfang Wang [1,2], Liyu Zhou[1], Zhen Ma [1], Mingxue He [1], Fang Wu [1,*] , Yunfei Liu [1]

[1] College of Information Science and Technology, Nanjing Forestry University, Nanjing 210037, People's Republic of China;
[2] Institution of Energy and Microstructure, Nanjing University of Science and Technology, Nanjing 210094, People's Republic of China;
* Correspondence: fangwu@mail.ustc.edu.cn (F. W.)

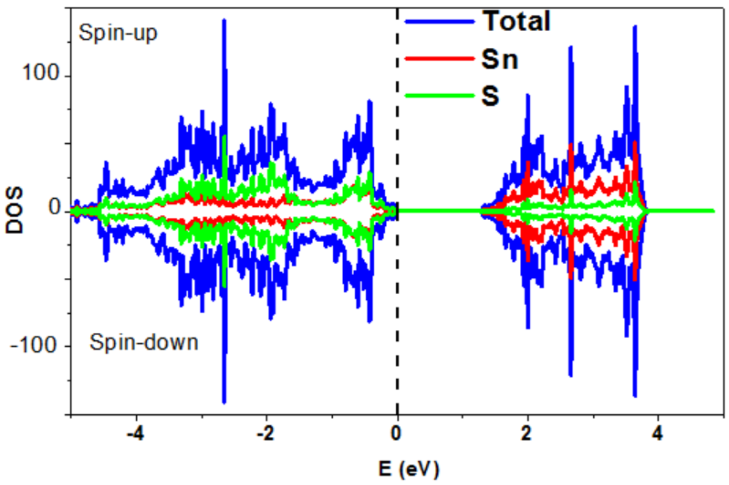

Figure S1. The calculated spin-resolved density of states of 2D SnS monolayer.

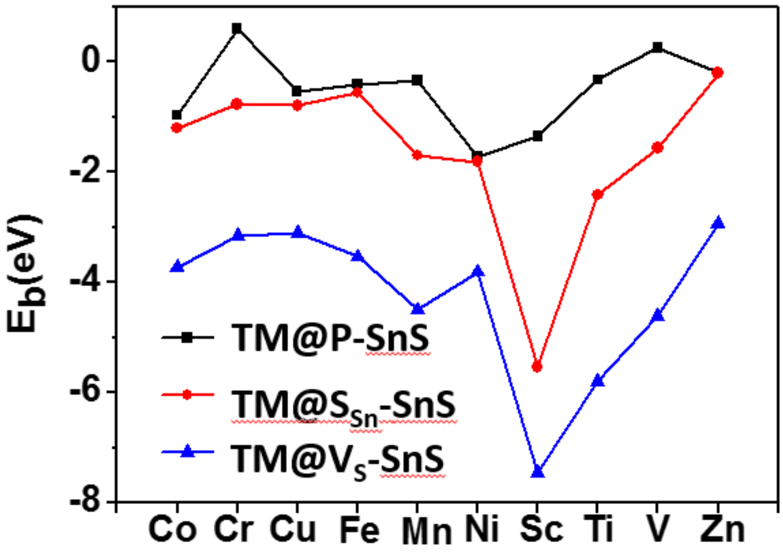

Figure S2. The calculated binding energy for each TM atom in 2D SnS monolayer.
